# Do wrist anatomical differences predispose to scapholunate ligament injury? A case-control radiographic study

**DOI:** 10.1186/s13018-025-06296-9

**Published:** 2025-09-22

**Authors:** Ebubekir Eravsar, Ali Gulec, Fatih Bilal Sezer, Ibrahim Ozkan, Ali Özdemir, Mehmet Ali Acar

**Affiliations:** 1 Department of Orthopedics and Traumatology, Selcuk University, 42130 Konya, Turkey; 2 Department of Orthopedics and Traumatology, Private Akademi Meram Hospital, 42090 Konya, Turkey

**Keywords:** Scapholunate, Risk factors, Radiographic measurements, Radial inclination, Ulnar variance, Lunate type, Wrist anatomy

## Abstract

**Purpose:**

Scapholunate interosseous ligament (SLIL) injury is the most common cause of carpal instability and may lead to scapholunate advanced collapse if untreated. While several anatomical wrist variations have been implicated in other wrist pathologies, limited studies have explored their role in SLIL injuries. This study aimed to compare specific anatomical parameters on wrist radiographs between patients with arthroscopically confirmed SLIL injuries and healthy individuals to identify potential anatomical predispositions.

**Methods:**

This study analyzed bilateral wrist radiographs of 87 patients who underwent arthroscopic dorsal capsulodesis for SLIL injuries between 2010 and 2023. A control group of 87 asymptomatic individuals with normal wrist radiographs was also included. Standardized anteroposterior and lateral wrist X-rays were collaboratively evaluated by three orthopedic surgeons. Parameters measured included radial inclination (RI), lunate fossa inclination (LFI), ulnar variance (UV), lunate tilting angle (LTA), lunate uncovering index (LUCI), carpal height ratio (CHR), palmar tilting angle (PTA), and lunate morphology. Group comparisons were performed, and a multiple logistic regression analysis was conducted using variables found to be significant in univariate analysis to identify independent anatomical predictors of SLIL injury.

**Results:**

There were no significant differences between the SLIL-injured patient group and the control group in terms of gender, age, and side (*p* > 0.05). RI (*p* < 0.001) and LFI (*p* = 0.016) were significantly lower, while LTA (*p* < 0.001) was significantly higher in the SLIL-injured patient group. Multiple logistic regression analysis revealed that lower RI (OR: 0.853, 95% CI: 0.769–0.946; *p* = 0.003) and higher LTA (OR: 1.126, 95% CI: 1.052–1.204; *p* = 0.001) were independently associated with SLIL injury. LFI did not remain significant in the final model. No significant differences were observed in UV, LUCI, PTA, CHR, or lunate type between groups.

**Conclusion:**

Low RI and increased LTA may represent anatomical risk factors for SLIL injury. Other parameters were not associated with an increased risk of injury. This study was not designed to establish a radiological diagnosis of SLIL injury; rather, it demonstrates that SLIL injuries may be influenced by individual anatomical variations. Further large scale studies are needed to validate these findings and to better understand the anatomical contributions to SLIL injury susceptibility.

## Introduction

The scapholunate interosseous ligament (SLIL) injury is considered the most common cause of carpal instability [1–3]. Clinically, SLIL injury can lead to complaints such as pain localized to the periscaphoid area of the affected wrist, clicking, and a decrease in grip strength and range of motion [4]. If left untreated, instabilities caused by SLIL injury may progress, ultimately leading to a staged osteoarthritic condition of the wrist known as scapholunate advanced collapse [5, 6]. While the SLIL is the primary stabilizer of the scapholunate joint, other ligaments also serve as secondary stabilizers to maintain its stability. The ligamentous structure of the SLIL and its anatomical relationship with surrounding ligaments in scapholunate joint stability have been extensively discussed in the literature [7].

Variations in wrist anatomy and their relationship with wrist disorders are among the intriguing topics in orthopedics and have been examined in the etiopathogenesis of many diseases. For instance, radial inclination has been associated with certain wrist problems, and it has been suggested that a flatter radial inclination may be linked to Kienböck’s disease [8]. Similarly, a negative ulnar variance has been linked to Kienböck’s disease [9], while a positive ulnar variance is known to be associated with ulnar impaction syndrome [10, 11]. Another evaluation classifies the lunate into different types based on the shape of its distal articulation. These types are reported to have distinct mechanical properties, and it has been suggested that the severity of carpal instabilities, the risk of ulnar impaction syndrome, and the likelihood of osteoarthritis may vary depending on these types [12, 13]. However, there are limited studies in the literature on anatomical variations and risk factors associated with SLIL injury [14, 15].

The mechanism of scapholunate injury typically occurs as a result of a fall onto an outstretched hand [16, 17]. However, despite falling on an outstretched hand, SLIL injury is not observed in every individual. Therefore, we believe that anatomical differences in patients’ wrists may influence the likelihood of SLIL injury. In this study, radiographic measurements of wrists in patients with arthroscopically confirmed SLIL injuries were compared with those of normal individuals. The study aims to identify potential anatomical variations that might increase the likelihood of developing an SLIL injury.

## Methods

This study was designed as a retrospective, non-paired case-control study. Ethical approval was obtained from the Selcuk University Local Ethics Committee (Date: 11.03.2025, Number: 2025/145). The study was reported in accordance with the STROBE (Strengthening the Reporting of Observational Studies in Epidemiology) guidelines. Data were collected from the hospital’s electronic archives. The study was conducted in the orthopaedics department of a university hospital. The department includes a hand surgery unit offering subspecialty training and is recognized as a regional referral center for complex hand surgery cases.

Between 2010 and 2023, a total of 158 patients who underwent dorsal capsulodesis for SLIL injury were identified from the clinical archives. Of these, 29 patients with a history of previous fracture, surgery or arthritic changes involving the wrist were excluded. Subsequently, 21 patients with concomitant lunotriquetral interosseous ligament (LTIL) injury, 17 patients with triangular fibrocartilage complex (TFCC) injury, and 4 patients with combined SLIL, LTIL, and TFCC injuries were also excluded. The remaining 87 patients were included in the study. Bilateral wrist radiographs of these 87 patients were analyzed. The injury mechanism was a fall in 59 patients and a punch in 6 patients, while 22 patients could not remember the mechanism of injury. As a control group, wrist X-rays of 87 healthy individuals were analyzed. All individuals in the control group were patients who presented to our orthopedic clinic after an acute wrist trauma, had no detected pathology on their radiographs, and remained asymptomatic during follow-up. During wrist arthroscopy performed on the operated patients, a systematic examination was carefully conducted. The SLIL and LTIL were probed from both the radiocarpal and midcarpal portals to assess fiber continuity, tension, and the presence of any step-off or gap. The TFCC was evaluated from the radiocarpal portal for the integrity of its central and peripheral components. Any partial or complete tear, laxity, or abnormal mobility was assessed. The SLIL injury was arthroscopically repaired using the dorsal capsulodesis technique with 3 − 0 polydioxanone sutures.

In our clinic, anteroposterior and lateral radiographs are obtained in a standardized manner. AP radiographs are taken using the “zero position” method, with the shoulder in 90° abduction, the elbow in 90° flexion, and the forearm placed on the X-ray table. To prevent deviation, the third finger is aligned with the forearm. For lateral radiographs, care is taken to ensure that the radius, capitate, and third metacarpal are aligned. All radiographic measurements were performed collaboratively by three orthopedic surgeons. The surgeons discussed and agreed upon each measurement, and thus a consensus value was used for each parameter. All measurements were performed digitally using the hospital’s Picture Archiving and Communication System (PACS) software (Enlil PACS, Turkiye) available at our institution. The following measurements were taken:

### Radiological parameters

**Fig. 1:**
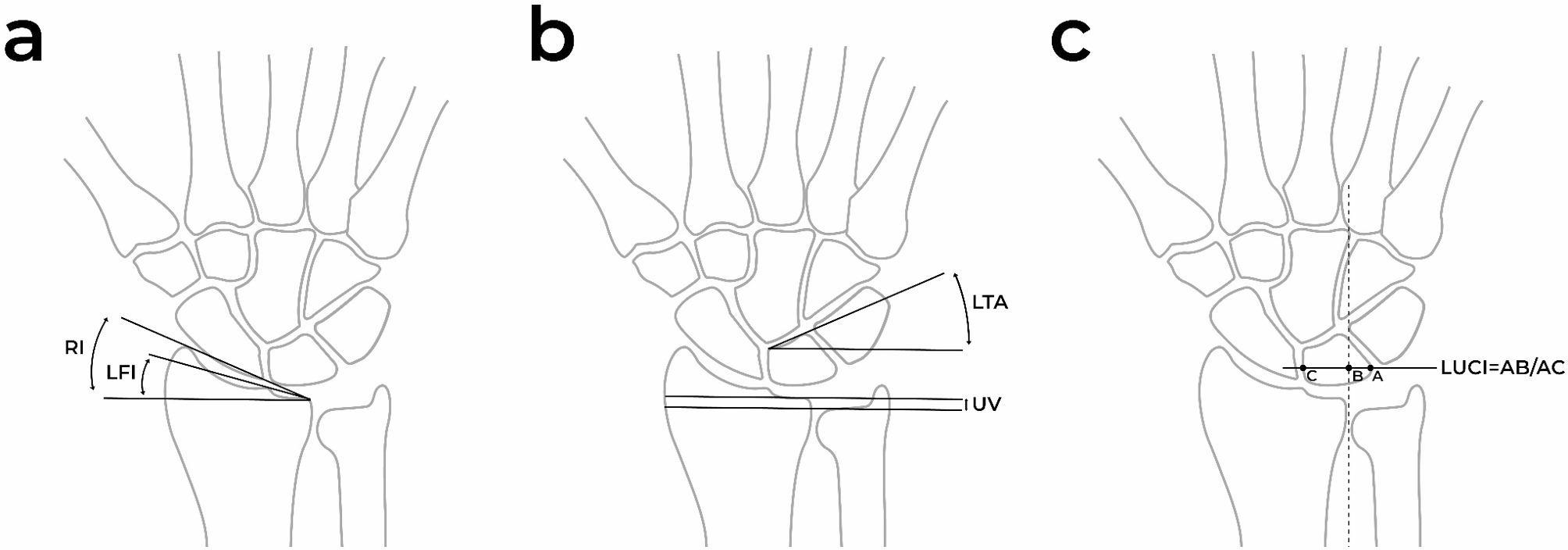
Illustration of measurements performed. **a** Radial Inclination (RI), Lunate Fossa Inclination (LFI) **b** Ulnar Variance (UV), Lunate Tilting Angle (LTA) **c** Lunate Uncovering Index (LUCI)

Radial Inclination (RI): The angle was determined between a line connecting the ulnar margin of the radial carpal surface to the radial styloid process and a perpendicular line to the radial axis [18] (Fig. 1a).

Lunate Fossa Inclination (LFI): The angle was measured between sclerotic line of the lunate fossa and a perpendicular line to the radial axis [19] (Fig. 1a).

Ulnar Variance (UV): A perpendicular line to the radial axis was drawn from the most ulnar point of the radial articular surface, and the distance to the distal cortex of the ulna was measured. In positive ulnar variance, the ulnar articular surface is located more distally relative to the radius, whereas in negative ulnar variance, it is positioned more proximally [20] (Fig. 1b).

Lunate Tilting Angle (LTA): The angle between the lunate’s baseline and a perpendicular line to the radial axis was recorded [15] (Fig. 1b).

Lunate Uncovering Index (LUCI): It was calculated as the ratio between the uncovered portion of the lunate along a line perpendicular to the longitudinal axis of the radial side of the distal radioulnar joint (DRUJ) and the total projection of the lunate on the same line [15] (Fig. 1c).

**Fig. 2:**
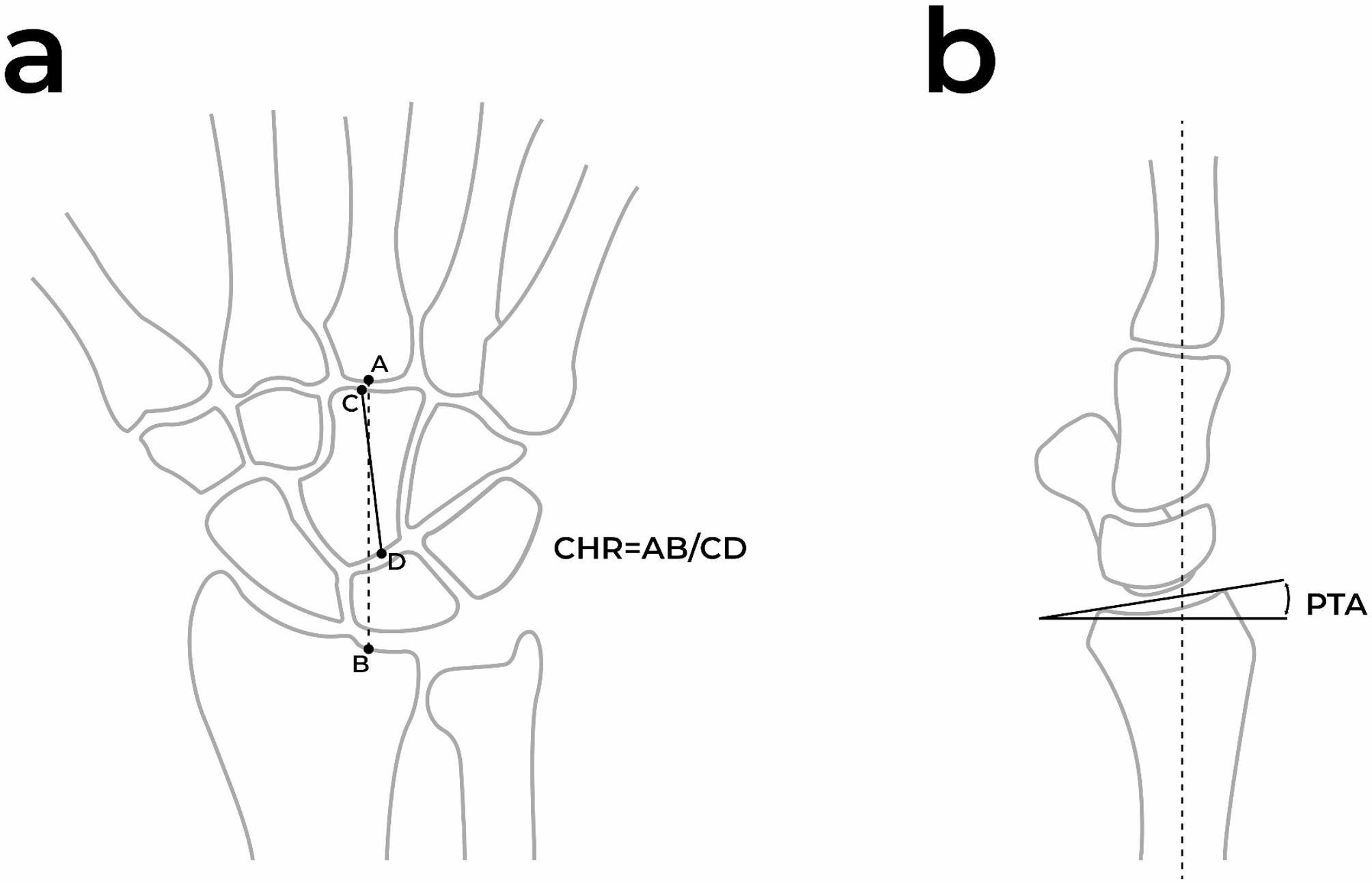
Illustration of measurements performed. **a** Carpal Height Ratio (CHR) **b** Palmar Tilting Angle (PTA)

Carpal Height Ratio (CHR): The ratio was calculated as the carpal height divided by the length of the capitate [21] (Fig. 2a).

Palmar Tilting Angle (PTA): The angle was measured between a line connecting the dorsal and palmar edges of the radial carpal surface and a perpendicular line to the radial axis [15] (Fig. 2b).

**Fig. 3:**
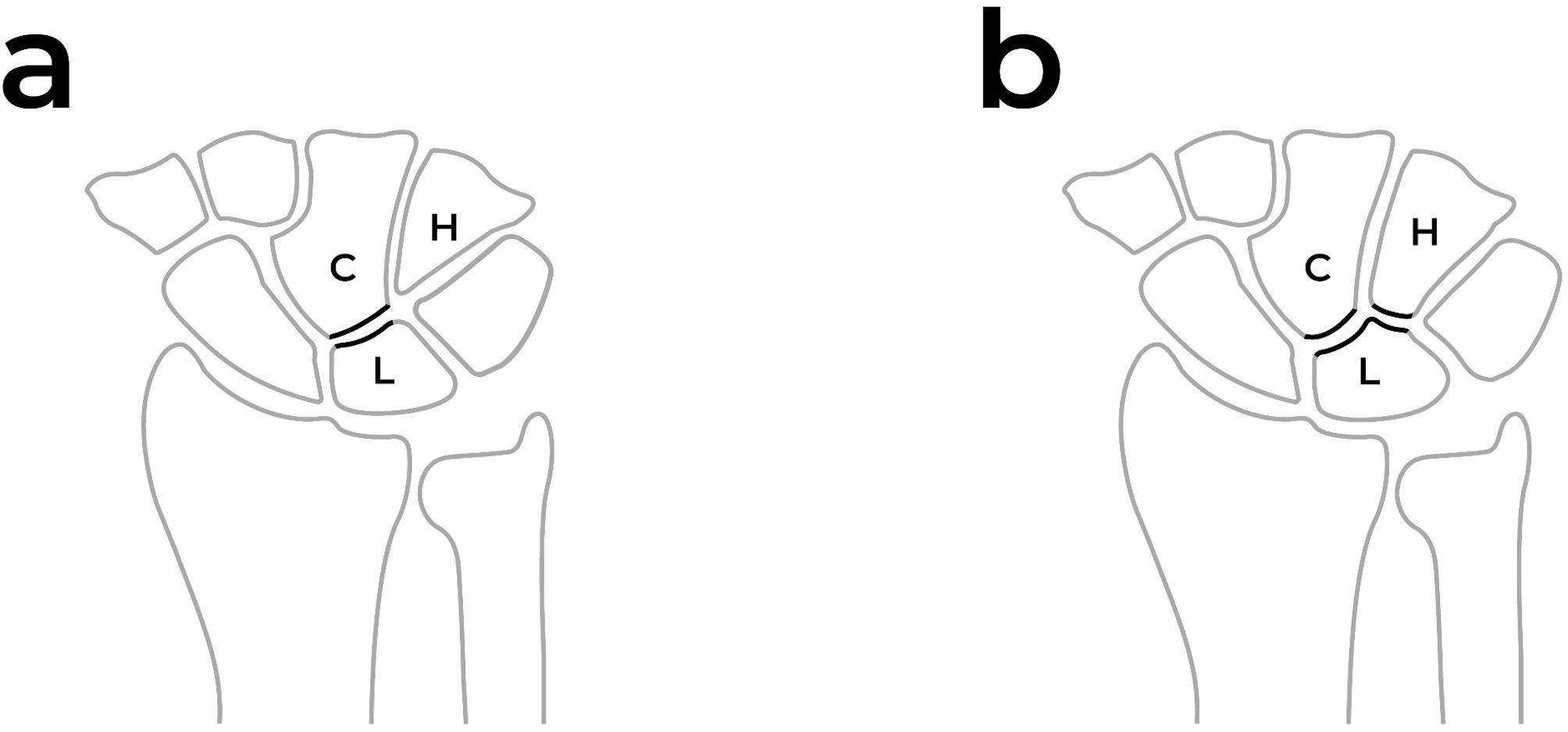
Depiction of Type I and Type II Lunate on the Illustration **a** Type I features a single distal facet that articulates solely with the capitate. **b** In contrast, Type II possesses an additional articular surface, enabling articulation with both the capitate and the hamate (C: capitate, L: lunate, H: Hamate)

Lunate Type: Classified based on its morphological variations. Type I lunate has a single distal facet that articulates with the capitate (Fig. 3a). Type II lunate has an additional articulation surface, allowing it to articulate with both the capitate and the hamate [22] (Fig. 3b). In cases where it was not possible to clearly distinguish between Type I and Type II, the lunate was classified as “intermediate” [23–25].

In the SLIL-injured patient group, measurements of radial inclination, lunate fossa inclination, ulnar variance, and palmar tilting angle were taken from the affected side, assuming that these parameters would not be influenced by the SLIL injury itself. Conversely, carpal height ratio, lunate uncovering index, lunate tilting angle, and lunate type were evaluated on the unaffected side to avoid potential misinterpretation due to the SLIL injury. According to the literature, in cases of unilateral wrist disorders, normal wrist measurements can be used as a reference for carpal height and carpal angle assessments; however, they are not considered reliable references for radial inclination, palmar tilting angle, and ulnar variance [26]. Comparisons of radiological measurements were made between the groups.

Statistics.

Assumptions of normality were checked with the Kolmogorov-Smirnov and Levene tests. For normally distributed data, the Independent Samples T-Test was used, while the Mann-Whitney U test was applied for non-normally distributed data. To reveal associations between the categorical data, the Chi-square test was conducted. Significance levels were set at *p* ≤ 0.05. Furthermore, variables that showed statistical significance in univariate analysis were included in a multiple logistic regression model using the Backward Wald variable selection method to identify independent predictors of SLIL injury. Odds ratios (ORs) with 95% confidence intervals (CIs) were reported, and statistical significance was set at *p* ≤ 0.05. All statistical analyses were carried out using SPSS 22 software (IBM, Armonk, NY, USA).

## Results

The median age of the SLIL-injured patient groupwas 33 (range, 18 to 55), while the median age of the control group was 35 (range, 18 to 59). 46% of the SLIL-injured patient group were female, and similarly, 47% of the control group were female. There were no significant differences between the SLIL-injured patient group and the control group in terms of gender (*p* = 0.879), age (*p* = 0.841), and side (*p* = 0.544) (Table 1). Radiological measurements showed that RI (*p* < 0.001) and LFI (*p* = 0.016) angles were lower in the SLIL-injured patient group compared to the control group. Additionally the LTA was higher in SLIL-injured patient group (*p* < 0.001). No significant difference was found between the SLIL-injured patient group and control group in terms of CHR (*p* = 0.118), UV (*p* = 0.660), LUCI (*p* = 0.720), and PTA (*p* = 0.484). In the SLIL-injured patient group, Type I lunate was observed in 26 individuals, Type II lunate in 40 individuals and intermediate lunate in 21 individuals; in the control group, Type I lunate was observed in 29 individuals, Type II lunate in 38 individuals and intermediate lunate in 20 individuals. No significant difference was found between the two groups in terms of lunate type (*p* = 0.216) (Table 1). In the multiple logistic regression model, lower RI (OR: 0.853, 95% CI: 0.769–0.946; *p* = 0.003) and higher LTA (OR: 1.126, 95% CI: 1.052–1.204; *p* = 0.001) were independently associated with SLIL injury (Table 2). LFI did not remain a significant predictor in the final model.

**Table 1:** Comparison of gender, side, age and radiological measurements by groups

	SLIL-injured patient group (*n* = 87)			Control group (*n* = 87)			*p*
	n (%)	Mean ± SD	Median (min to max)	n (%)	Mean ± SD	Median (min to max)	
Gender	40 female (%46) 47 male (%54)			41 female (%47) 46 male (%53)			0.879 *
Side	42 right (%48) 45 left (%52)			46 right (%53) 41 left (%47)			0.544 *
Age		34.27 ± 10.51	33 (18 to 55)		35.09 ± 11.97	35 (18 to 59)	0.841 **
Radial Inclination (RI)		20.97 ± 3.42	20 (13 to 30)		22.79 ± 2.93	23 (16 to 30)	< 0.001 **
Lunate Fossa Inclination (LFI)		10.87 ± 2.84	11 (2 to 18)		11.82 ± 2.70	12 (5 to 18)	0.016 **
Ulnar Variance (UV)		0.19 ± 1.70	0 (-3 to 4)		0.08 ± 1.52	0 (-3 to 4)	0.660 **
Carpal Height Ratio (CHR)		1.49 ± 0.11	1.48 (1.24 to 2.00)		1.52 ± 0.09	1.52 (1.32 to 1.77)	0.118 ***
Lunate Uncovering Index (LUCI)		0.28 ± 0.09	0.29 (0.08 to 0.53)		0.27 ± 0.11	0.28 (0.07 to 0.47)	0.720 **
Palmar Tilting Angle (PTA)		9.69 ± 3.91	10 (0 to 18)		9.18 ± 4.76	9 (0 to 19)	0.484 **
Lunate Tilting Angle (LTA)		20.42 ± 5.56	20 (10 to 36)		16.98 ± 4.89	18 (5 to 31)	< 0.001 **
Lunate Type	26 Type I (%30) 40 Type II (%46) 21 Intermediate (%24)			29 Type I (%33) 38 Type II (%44) 20 Intermediate (%23)			0.887 *

* Chi-square test

** Mann-Whitney U test

*** T-test

min, Minimum; max, Maxiumum; n, Number; SD, Standard Deviation; SLIL, Scapholunate Interosseous Ligament

**Table 2:** Multiple logistic regression analysis

	*Odds Ratio ( %95 CI )*	*p value*
Radial Inclination (RI)	0.853 (0.769–0.946)	**0.003**
Lunate Tilting Angle (LTA)	1.126 (1.052–1.204)	**0.001**
Lunate Fossa Inclination (LFI)	1.002 (0.863–1.163)	0.980

## Discussion

The mechanism of scapholunate injury typically occurs through hyperextension, ulnar deviation, and supination when falling onto an outstretched hand [16, 17]. During the impact of the hand with the ground, stress transmitted from the more distal capitate is conveyed to the scapholunate joint, causing the capitate to intrude into this joint [4]. As a result, the scaphoid moves dorsally and radially, while the lunate moves ulnarly and volarly, forcing them in opposite directions. This leads to a tear in the SLIL, starting from the volar edge and progressing towards the dorsal part [27]. However, despite falling on an outstretched hand, SLIL injury is not observed in every individual. This suggests that additional factors, such as individual anatomical variations, may play a role in susceptibility to injury. The aim of this study was to identify potential anatomical variations that might increase the risk of SLIL injury through radiographic evaluation.

RI is one of the anatomical differences identified in this study. It was found that patients with SLIL injuries had lower RI and lower LFI compared to normal individuals. In a similar study by Thienpoint et al., findings parallel to ours indicated that lower RI and lower LFI are potential risk factors for scapholunate injury [15]. However, unlike that study, our analysis included a multiple logistic regression model, which revealed that LFI did not independently contribute to the risk in the final model, whereas lower RI was found to be an independent risk factor for SLIL injury. During a fall, as the wrist moves into ulnar deviation, the scaphoid transitions from a flexion-radial deviation position to an extension-ulnar deviation position. With decreased RI, the scaphoid loses its vertical position, thereby increasing stress on the scapholunate ligament [28]. Additionally, during a fall, the transmission of load from the capitate may pass through the scapholunate interval. If the radius has a lower inclination, it may not provide sufficient support to the scaphoid via the radial styloid, making it easier for the scaphoid to shift radially. A similar phenomenon can be considered on the ulnar side. If the distal ulna is short, the load transmitted from the capitate to the scapholunate interval may cause the lunate, which lacks sufficient support from the distal ulna, to shift easily into the soft area where the TFCC is located on the ulnar side. This condition may lead to an increase in the separating forces acting on the SLIL, ultimately causing ligament rupture. Supporting this theory, the effect of UV on SLIL injuries has been investigated in previous studies. Czitrom et al. found that scapholunate injuries were associated with negative UV [14]. However, in our study, no relationship between UV and SLIL injury was identified. Additionally, De Smet et al. reached a similar conclusion, finding that UV had no effect on this type of injury [29].

Another measurement examined in this study was the LTA, which was found to be higher in the SLIL-injured patient group compared to the control group. This finding can also be explained by the injury mechanism. The load causing SLIL damage is transmitted through the capitate, leading to the separation of the scapholunate joint. As the distal articular surface of the lunate changes from a horizontal to a vertical position, the capitate can more easily slide into the scapholunate interval. This may increase the likelihood of SLIL injury. Additionally, this study examined the relationship between CHR and SLIL injury. Lower CHR ratios indicate that the capitate occupies a larger portion of the wrist. Therefore, we hypothesized that patients with lower CHR values would have a higher risk of SLIL injury. However, our measurements did not show any significant difference between patients with SLIL injuries and the control group.

One of the important anatomical differences in the wrist is the shape of the lunate and its proximal articular surfaces. The lunate is considered the keystone of the wrist. Viegas classified the lunate into two types, Type I and Type II, based on its midcarpal articulation. Type I lunates have a single distal facet that articulates solely with the capitate, whereas Type II lunates have an additional facet that articulates with the hamate as well [22]. In some borderline cases between the two types, the lunate morphology may be classified as intermediate or equivocal [23–25]. Greater scaphoid translation has been observed in wrists with a Type I lunate compared to those with a Type II lunate [25]. Some studies suggest that lunate morphology is associated with the severity of scapholunate injuries and that SLIL injuries tend to be more severe in Type I lunates [12]. Furthermore, in cases where an SLIL tear is present, patients with a Type I lunate are reported to have a higher likelihood of developing DISI [30]. The underlying rationale for these findings is that the presence of a second articular facet in Type II lunates may provide additional stability, potentially preventing the progression to DISI even if an SLIL tear occurs. While previous studies have focused on the severity and progression of SLIL injuries in affected patients, our study takes a different approach. In our study, we investigated whether lunate type differs between patients with SLIL injuries and normal individuals. No significant difference was found between the two groups. These findings suggest that the presence or absence of an additional articular facet with the hamate may not play a role in the occurrence of SLIL injuries.

On standard radiographs, widening of the scapholunate interval is a well-recognized radiological sign of SLIL injuries. However, current evidence suggests that an isolated SLIL injury alone is often insufficient to produce a static instability and diastasis visible on routine radiographs. This radiographic widening typically develops following injury to the primary stabilizer, the SLIL, as a result of weakening or damage to the secondary stabilizers, the dorsal intercarpal ligament and the dorsal radiocarpal ligament [31–33]. Therefore, although scapholunate diastasis remains one of the most characteristic findings, its absence does not exclude the diagnosis of an SLIL injury. Injuries that cannot be detected on standard or stress radiographs but are identified through advanced imaging or wrist arthroscopy are referred to as predynamic (occult) instability [4]. One of the most important advanced imaging techniques is MRI. However, MRI imaging alone may be insufficient for a definitive diagnosis [34]. Wrist arthroscopy has become one of the most important techniques for diagnosing and treating SLIL injuries, due to the magnification provided by the scope and the ability to examine the ligament using probes [3]. In our study, the diagnosis and treatment of SLIL injuries were examined in patients who underwent wrist arthroscopy. This allowed for a definitive diagnosis of SLIL injury and the exclusion of additional injuries such as LTIL and TFCC.

This study has some important limitations. The most significant limitation is the small sample size. Additionally, the external validity of our findings may be limited. All patients included in the study were treated at a single tertiary center, and the majority of participants belonged to a relatively homogenous population in terms of ethnicity and geographic location. Therefore, caution should be exercised when extrapolating these results to other populations with different demographic or anatomical characteristics. To more effectively study the impact of anatomical variations on SLIL injuries and to validate the generalizability of these findings, future multi-center studies involving larger and more diverse populations are needed. Another important limitation is the heterogeneity in injury mechanisms among SLIL-injured patients, with many having sustained their injuries from falls onto the hand. SLIL injuries can develop due to different anatomical characteristics depending on the injury mechanism. In such cases, the hypotheses we established may not be sufficiently supported. Another limitation is that, since not all possible structural variations of the wrist could be evaluated in this study, the generalizability of our results is limited and should be supported by further research. In this study, radiological measurements were performed collaboratively by three orthopedic surgeons, and consensus was reached for each measurement. However, this may be considered a limitation, as independent assessments followed by an interobserver reliability analysis could have further strengthened the methodological rigor of the study. Although this study investigates the predisposition to SLIL injuries based on anatomical features identified through direct radiographs, it should be noted that direct radiographs are insufficient for detecting SLIL injuries. Therefore, the findings from this study were not intended to diagnose SLIL injuries based solely on direct radiographs.

In conclusion, this study compared the wrist anatomical differences between patients with SLIL injuries and healthy individuals radiologically, and attempted to identify differences that might increase the likelihood of this injury. The findings of the study suggest that low RI and high LTA may be potential risk factors for SLIL injuries. However, no significant differences were found in other anatomical parameters such as UV, PTA, LUCI, and CHR. Our results also suggest that lunate morphology is not a determining factor in the development of SLIL injuries. This study does not aim to diagnose SLIL injuries using direct radiographs; rather, it demonstrates that SLIL injuries may be influenced by anatomical differences among individuals. However, considering the limited sample size, heterogeneity of injury mechanisms, and the fact that structural variations could not be comprehensively accounted for, future studies with larger patient groups, more diverse populations, and detailed anatomical assessments are needed to clarify these relationships in more detail.

## Data Availability

The datasets generated and/or analysed during the current study are not publicly available due to the fact that they involve patient-related information obtained from the hospital but are available from the corresponding author on reasonable request.
